# The molecular mechanism of acute lung injury caused by *Pseudomonas aeruginosa*: from bacterial pathogenesis to host response

**DOI:** 10.1186/2052-0492-2-10

**Published:** 2014-02-18

**Authors:** Teiji Sawa

**Affiliations:** Department of Anesthesiology, Kyoto Prefectural University of Medicine, 465 Kajii-cho Kamigyo, Kyoto, 602-8566 Japan

**Keywords:** Acute lung injury, Pneumonia, *Pseudomonas aeruginosa*, Type III secretion system, ExoU

## Abstract

*Pseudomonas aeruginosa* is the most common gram-negative pathogen causing pneumonia in immunocompromised patients. Acute lung injury induced by bacterial exoproducts is associated with a poor outcome in *P. aeruginosa* pneumonia. The major pathogenic toxins among the exoproducts of *P. aeruginosa* and the mechanism by which they cause acute lung injury have been investigated: exoenzyme S and co-regulated toxins were found to contribute to acute lung injury. *P. aeruginosa* secretes these toxins through the recently defined type III secretion system (TTSS), by which gram-negative bacteria directly translocate toxins into the cytosol of target eukaryotic cells. TTSS comprises the secretion apparatus (termed the injectisome), translocators, secreted toxins, and regulatory components. In the *P. aeruginosa* genome, a pathogenic gene cluster, the exoenzyme S regulon, encodes genes underlying the regulation, secretion, and translocation of TTSS. Four type III secretory toxins, namely ExoS, ExoT, ExoU, and ExoY, have been identified in *P. aeruginosa.* ExoS is a 49-kDa form of exoenzyme S, a bifunctional toxin that exerts ADP-ribosyltransferase and GTPase-activating protein (GAP) activity to disrupt endocytosis, the actin cytoskeleton, and cell proliferation. ExoT, a 53-kDa form of exoenzyme S with 75% sequence homology to ExoS, also exerts GAP activity to interfere with cell morphology and motility. ExoY is a nucleotidal cyclase that increases the intracellular levels of cyclic adenosine and guanosine monophosphates, resulting in edema formation. ExoU, which exhibits phospholipase A2 activity activated by host cell ubiquitination after translocation, is a major pathogenic cytotoxin that causes alveolar epithelial injury and macrophage necrosis. Approximately 20% of clinical isolates also secrete ExoU, a gene encoded within an insertional pathogenic gene cluster named *P. aeruginosa* pathogenicity island-2. The ExoU secretory phenotype is associated with a poor clinical outcome in *P. aeruginosa* pneumonia. Blockade of translocation by TTSS or inhibition of the enzymatic activity of translocated toxins has the potential to decrease acute lung injury and improve clinical outcome.

**Electronic supplementary material:**

The online version of this article (doi:10.1186/2052-0492-2-10) contains supplementary material, which is available to authorized users.

## Introduction

*Pseudomonas aeruginosa* is one of the most common gram-negative pathogens causing pneumonia in immunocompromised patients [1–4]. Ventilated patients are at particularly high risk of developing *P. aeruginosa* pneumonia [5, 6], and the mortality rate of ventilator-associated pneumonia (VAP) due to *P. aeruginosa* is significantly higher than that due to other pathogens [7–9]. Some *P. aeruginosa* strains possess the ability to destroy the integrity of the alveolar epithelial barrier, causing rapid necrosis of the lung epithelium and bacterial dissemination into the circulation [10, 11]. Understanding the mechanism by which virulent strains of *P. aeruginosa* cause acute lung injury is critical for preventing subsequent sepsis and death. The present review summarizes the progress and explains the mechanisms causing acute lung injury and sepsis, focusing on the type III secretion system (TTSS) of *P. aeruginosa*.

## Review

### Acute lung epithelial injury caused by *P. aeruginosa*

#### Acute lung injury in animal models

*P. aeruginosa* secretes various toxic exoproducts (Table 1). Investigation of the toxic exoproducts of *P. aeruginosa* with major roles in acute lung injury began in the late 1980s. In animal models, acute lung epithelial injury was quantified through the measurement of bidirectional protein movement across the lung epithelial barrier [12–14]. In this model, the airspace instillation of live *P. aeruginosa* resulted in increased movement of the alveolar tracer into the vascular compartment, a twofold increase in the vascular tracer in the airspace, and a significant reduction in liquid clearance by the lung, while instillation of *Escherichia coli* endotoxin did not cause lung epithelial injury. These early animal experiments initiated the search for a major virulence factor responsible for acute lung epithelial injury among the exoproducts of *P. aeruginosa*[15, 16].

**Table 1:** **The major toxic exoproducts of** ***Pseudomonas aeruginosa***

Exoproducts	Locus ID, PA number	Effect on host	Secretion type	Regulation system
Exotoxin A	*toxA*, PA1148	Antiphagocytic, cytotoxic	Type II	(LasR-LasI quorum sensing)
Exoenzyme S	*exoS*, PA3841	Antiphagocytic, cytotoxic	Type III	ExsA-activated type III system
Elastase (LasA, LasB)	*lasA*, PA1871	Elastolytic activity	Type II	LasR-LasI quorum sensing
	*lasB*, PA3724			
Alkaline proteinase	*aprA*, PA1249		Type I	LasR-LasI quorum sensing
Phospholipase C	*plcH*, PA0844	Disturbance of membrane lipid metabolism	Type II	Inorganic phosphate
	*plcN*, PA3319			

#### Discovery of a major cytotoxin: ExoU

The *P. aeruginosa* toxin exoenzyme S was identified in the late 1970s as an ADP-ribosyltransferase distinct from exotoxin A [17, 18]. Early studies revealed that the exoenzyme S-positive phenotype correlated with increased virulence in lung infections and burn wounds [19–24]. The protein transcriptional regulator ExsA was found to regulate the production of exoenzyme S and co-regulated proteins [25–27]. PAO-S21, an insertional mutant of transposon Tn501 in the *exsA* gene of *P. aeruginosa*, is exoenzyme S-deficient [15, 19]. PAO-S21 infection did not result in altered protein flux across the alveolar epithelial barrier [15]. Based on these findings, exoenzyme S, or an unknown exoenzyme S-related toxin regulated by ExsA, was determined to play a major role in acute lung injury. Exoenzyme S activity was later determined to be the result of two highly homologous toxins, ExoS (a 49-kDa form of exoenzyme S) and ExoT (a 53-kDa form of exoenzyme S), encoded by two separate regions of the *P. aeruginosa* genome [28–31].

The virulent *P. aeruginosa* strain PA103, lacking the 49-kDa form of the exoenzyme S gene (*exoS*) but possessing the 53-kDa form (*exoT*), causes a high degree of acute injury [16]. Because the isogenic mutant lacking the 49-kDa form of exoenzyme S remained capable of causing acute lung injury in a rabbit model, it was initially considered possible that ExoT is the major factor underlying acute lung injury [16]. However, an isogenic mutant lacking ExoT remained capable of causing alveolar epithelial injury in a mouse model [32]. Thus, neither ExoT nor ExoS was the major virulence factor. PA103 was found to secrete a unique unknown 74-kDa protein, the production of which decreased with a transposon mutation in *exsA*. The gene encoding this 74-kDa protein was cloned, and a mutant missing this protein was created in PA103. PA103 lacking this 74-kDa protein failed to cause acute lung injury in our mouse model [33, 34]. This protein, regulated by ExsA, was named ExoU. Clinical isolates with a cytotoxic phenotype *in vitro* were found to express ExoU and cause acute epithelial injury in a mouse model [33]. Cytotoxic *P. aeruginosa* isolates were identified to possess *exoU*, while noncytotoxic isolates lacked the gene [33]. High cytotoxicity, severity of lung epithelial injury, and bacterial dissemination into the circulation appeared to show a high correlation with the *exoU* genotype [35, 36]. Therefore, it was concluded that the ability of *P. aeruginosa* to cause acute lung epithelial injury and sepsis is highly linked to the expression of ExoU, regulated by the transcriptional activator ExsA [33, 34].

### Type III secretion system

#### The secretion systems of gram-negative bacteria

Gram-negative bacteria, which have inner and outer bacterial membranes, use dedicated secretion systems to transport proteins synthesized to the outside environment. The secretion systems of gram-negative bacteria can be classified into six subtypes [37]. The type I secretion system is relatively simple, consisting of only a few proteins. Unlike proteins secreted by the type II secretion system, proteins secreted by the type I secretion system contain no signal sequence at their amino termini; instead, they contain domains at their carboxyl termini necessary for recognition by the type I secretion complex. The type II system conducts so-called *sec*-dependent secretion [38]. Proteins secreted by the type II system possess amino-terminal signal sequences of 16–26 residues [38].

**Figure 1:**
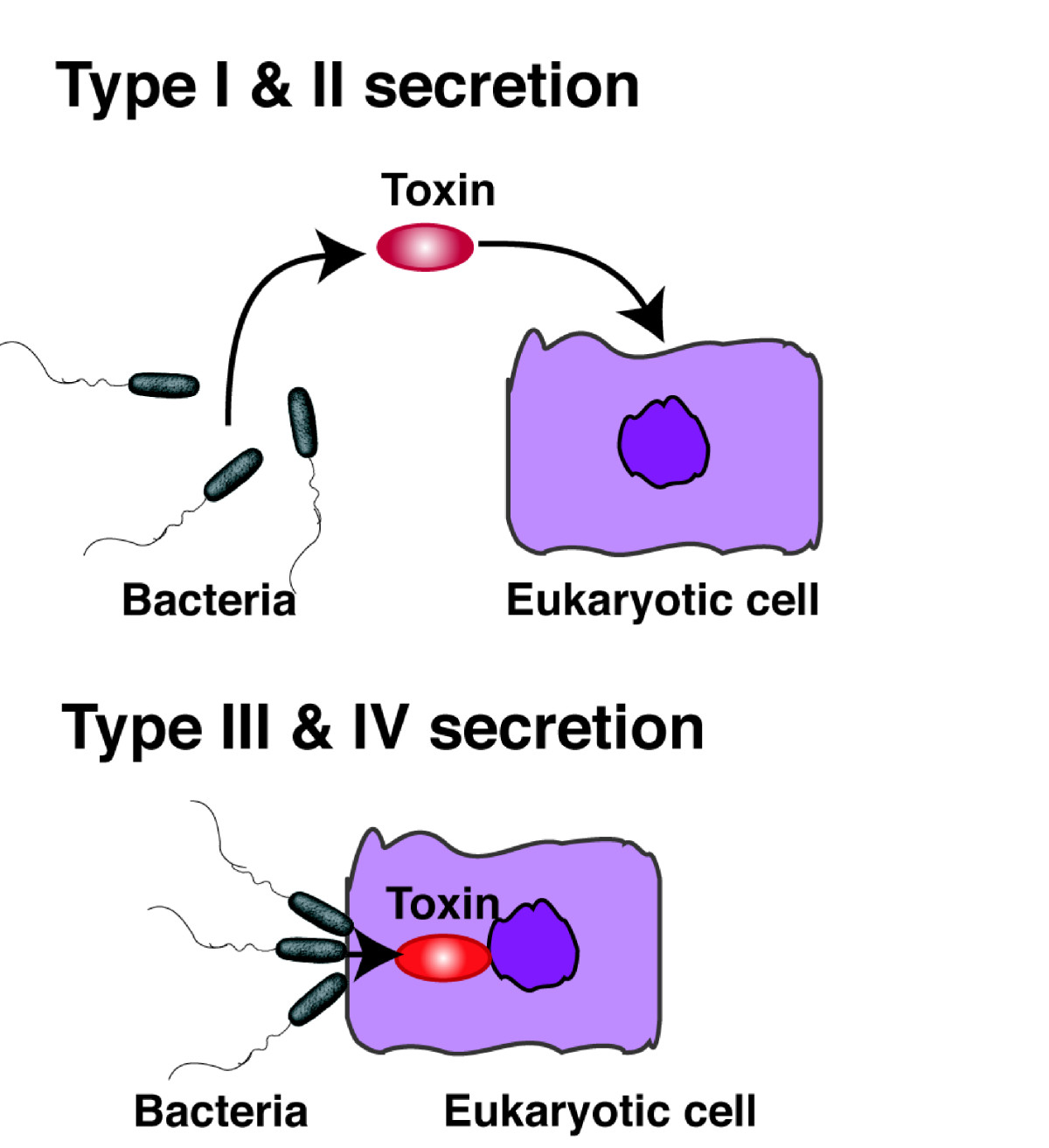
**Gram-negative bacterial protein secretion system.** In the type I and II secretion systems, bacteria secrete toxins into the extracellular space (*upper image*). In the type III and IV secretion systems, bacteria directly secrete toxins into the cytosol of target eukaryotic cells through the secretion apparatus (*lower image*).

The type III and IV secretion systems have been more recently defined (Figure 1). Recently, a high degree of association has been reported to exist between the type III and IV secretion systems and the pathogenesis of gram-negative bacteria [37, 39]. In both the secretion systems, bacteria directly deliver proteins into the cytosol of target eukaryotic cells [40]. Evolutionarily, TTSS is derived from flagella, while the type IV system is derived from a conjugational system [39, 41]. TTSS is utilized by most pathogenic gram-negative bacteria, including *Yersinia*, *Salmonella*, *Shigella*, *E. coli*, and *P. aeruginosa* (Table 2) [42]. TTSS functions as a molecular syringe, directly delivering toxins into the cytosol of cells [43]. The translocated toxins modulate eukaryotic cell signaling. All TTSSs studied till date share an important feature: the genes encoding this system are upregulated by direct contact between bacteria and host cells, with consequent direct delivery of bacterial virulence products (type III secretory toxins or effector molecules) into the host cell via the secretion and translocation apparatus [42]. In *P. aeruginosa*, exoenzyme S was initially thought to be secreted via the type II secretion pathway. However, based on the genomic homology to other gram-negative bacteria, this toxin and co-regulated toxins (ExoT, ExoU, and ExoY) were ultimately determined to be translocated as effector proteins into host cells via TTSS [44].

**Table 2:** **Type III secretion systems in animal-associated gram-negative bacteria**

Bacteria	Effect on host	Secreted proteins	Secretion apparatus
*Pseudomonas aeruginosa*	Cytotoxic, antiphagocytic	Exo, Pop	Psc
*Bordetella* spp.	Cytotoxic	Bop	Bsc
*Burkholderia pseudomallei*	Facilitates invasion, etc.	Bop	Bsa
*Chlamydia* spp.	Prevents microtubule assembly, etc.	Cop	Cds
Pathogenic *E. coli*	A/E lesion formation	Esp, Tir	Sep
*Salmonella* spp.	Bacterial entry, apoptosis	Sip, Sop	Inv, Prg, Spa, Sip
*Shigella* spp.	Bacterial entry, apoptosis	Ipa, VirA	Spa, Mxi
*Yersinia* spp.	Cytotoxic, antiphagocytic	Yop, Lcr	Ysc

#### Genomic organization of *P. aeruginosa* TTSS

**Figure 2:**
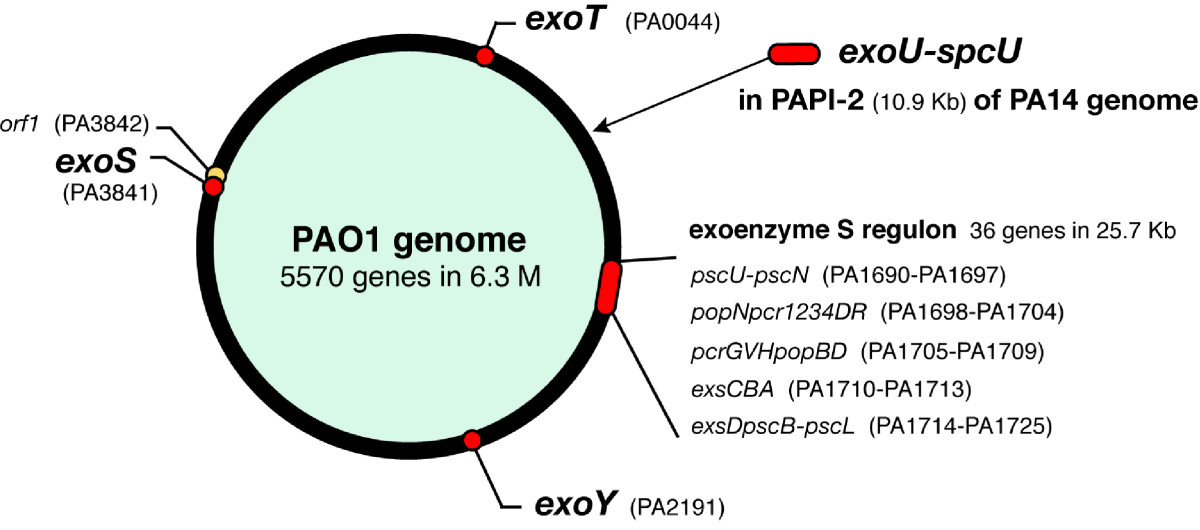
**The** ***Pseudomonas aeruginosa*** **genome and type III secretion regulon and toxin genes.** The genomic DNA of *P. aeruginosa* strain PAO1 was completely sequenced by the *Pseudomonas* Genome Project in 2000. Within the 6.3-Mb region, 5,570 open reading frames were found. The type III secretion regulatory region (25.5 kb) was found as a gene cluster and named the exoenzyme S regulon. It comprised five operons, including 36 genes for transcription (*exsA*-*exsD*), secretion apparatus (*pscB*-*pscU*), and translocation (*pcrGVHpopBD*). The genes of the type III secretory toxins *exoS*, *exoT*, and *exoY*, but not *exoU*, were scattered throughout the genome. The *exoU* gene was found to be located in an insertional pathogenic gene cluster named *P. aeruginosa* pathogenicity island-2 (PAPI-2) discovered in the virulent clinical strain PA14.

TTSS of *P. aeruginosa* is highly homologous to the prototypical *Yersinia* TTSS [45, 46]. The whole genome of *P. aeruginosa* strain PAO1 was sequenced by the *Pseudomonas* Genome Project and published in 2000 (Figure 2) [47]. It was found that the 25.6-kb genomic region, named the exoenzyme S regulon, encodes genes underlying the regulation, secretion, and translocation of TTSS [48]. Expression of these genes is under the regulation of the transcriptional activator protein ExsA, and ExsA itself is encoded by the *exsCBA* operon in the exoenzyme S regulon [28, 48].

In the genome of *P. aeruginosa* PAO1, three type III secretory toxins (excluding ExoU), co-regulated with the exoenzyme S regulon by ExsA, have been identified (Figure 2). These are ExoS (a 49-kDa form of exoenzyme S), ExoT (a 53-kDa form of exoenzyme S, also known as exoenzyme T), and ExoY [31, 49]. The genes encoding these type III secretory toxins (*exoS*, *exoT*, and *exoY*) are distributed in regions of the genome separate from the exoenzyme S regulon [47, 48]. Later, two distinct *P. aeruginosa* pathogenicity islands, PAPI-1 (108 kb) and PAPI-2 (11 kb), which are absent from the less virulent strain PAO1, were found in the highly virulent clinical strain PA14, and *exoU* was discovered in the PAPI-2 region of this strain [50, 51]. Approximately 20% of clinical isolates are more virulent; they possess *exoU*, but not *exoS*[52].

### The exoenzyme S regulon

#### Transcriptional activator ExsA

ExsA, encoded by the *exsCBA* operon (the trans-regulatory locus for exoenzyme S secretion) in the exoenzyme S regulon, is a transcriptional activator of the *P. aeruginosa* TTSS [48]. In the exoenzyme S regulon, ExsA regulates the transcription of five operons (*exsD*-*pscL*, *exsCBA*, *pscG*-*popD*, *popN*-*pcrR*, and *pscN*-*pscU*) encoding TTSS and the translocation machinery (Figure 2) [48]. Another four or five ExsA-binding sites have been found in the genome for the regulation of effector molecules (type III secretory toxins) and their chaperones [47].

#### Secretion apparatus

**Figure 3:**
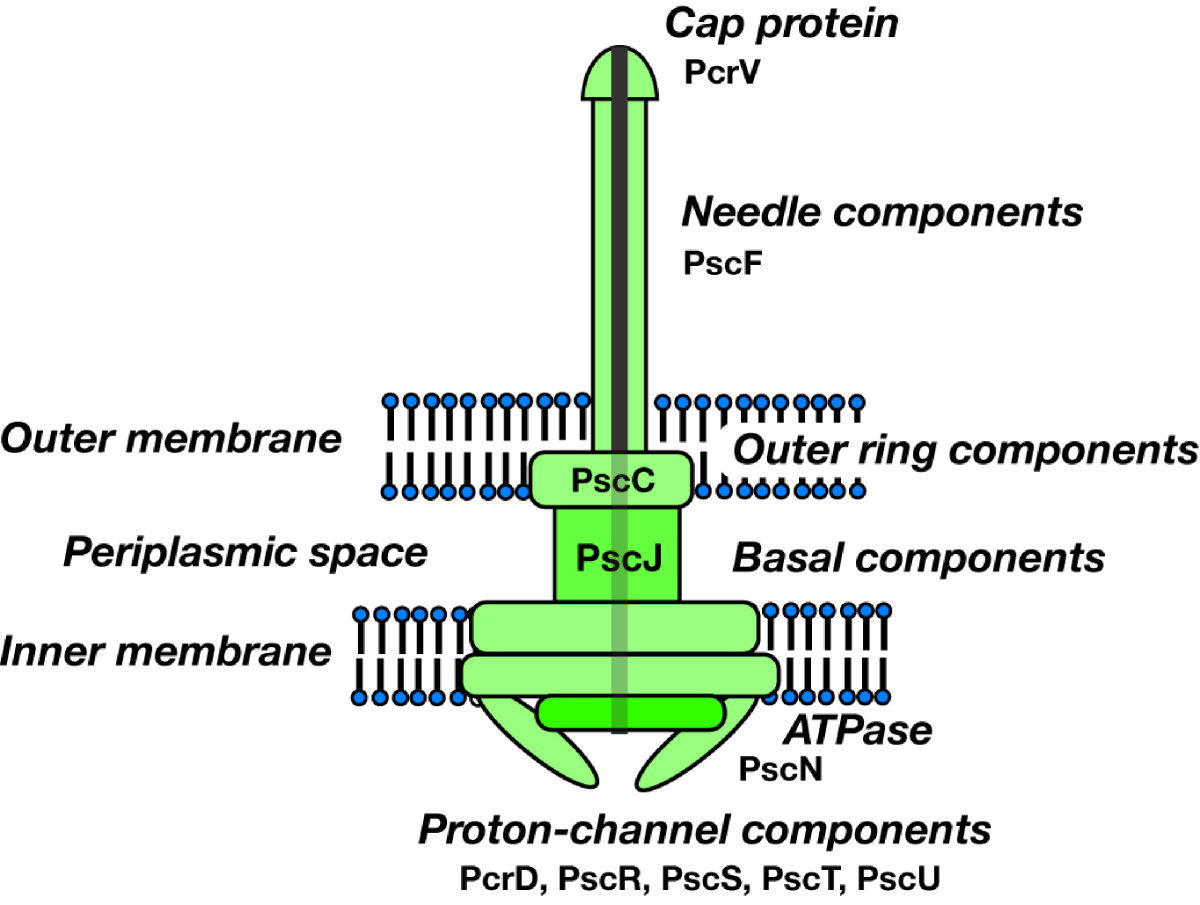
***Pseudomonas aeruginosa*** **type III secretory apparatus: the needle complex or injectisome.** The type III secretory apparatus comprises many protein components: a cap component, PcrV; a needle component, PscF; an outer ring component, PscC; and basal components, including PscJ, ATPase PscN, and others.

In TTSS, *secretion* describes the process by which toxins are transferred from the bacterial cytosol to the surrounding medium across the inner and outer bacterial membranes [43]. This process seems to require *secretion apparatus* involving many protein components (Figure 3). All known TTSSs in animals and pathogenic bacteria share a number of highly conserved core structural components. The TTSS-specific export apparatus is termed the needle complex in *Salmonella*[53, 54], *Shigella*[55], and *E. coli*[56]. This structure spans both the inner and outer membranes of the bacterial envelope and closely resembles the flagella basal body, further supporting the evolutionary relationship between the flagella and TTSS [41, 57].

In *Yersinia*, *ysc* genes in the Yop virulon largely encode components of TTSS, and *P. aeruginosa* possesses homologous *psc* genes in its exoenzyme regulon [45, 48]. Ysc proteins from *Yersinia ysc* genes and Psc proteins from *P. aeruginosa psc* genes are considered as components of their respective needle complexes because of their sequence homology to *Salmonella* Spa, Prg, and Inv; *Shigella* Spa and Mxi; and *E. coli* Esc proteins.

#### Translocators and V-antigen

In TTSS, *translocation*, which describes the process of direct toxin transfer into the eukaryotic cytosol across the eukaryotic plasma membrane, has been thoroughly investigated in *Yersinia*[45, 58–60]. In *P. aeruginosa*, the *pcrGVHpopBD* operon, under regulation by ExsA, encodes five proteins, namely PcrG, PcrV, PcrH, PopB, and PopD, homologous to *Yersinia* LcrG, LcrV, LcrH, YopB, and YopD, respectively (Table 3) [61, 62]. Translocation in the *P. aeruginosa* TTSS is mediated by PcrV, PopB, and PopD. In fact, in *P. aeruginosa*, isogenic mutants lacking *pcrV* or *popD* were unable to intoxicate eukaryotic cells [63, 64]. Historically, *Yersinia* LcrV was designated *Yersinia* V-antigen and thought to protect mice from lethal infections with *yersiniae* strains [65, 66]. PcrV (*P. aeruginosa* V-antigen) corresponds to the *Yersinia* V-antigen LcrV. Antibodies against LcrV and PcrV are likely to block type III protein translocation by interfering with pore formation by LcrV/YopB/YopD and PcrV/PopB/PopD, respectively [63, 64].

**Table 3:** **Proteins required for the translocation of** ***Pseudomonas aeruginosa*** **Exo effectors**

Protein	PA number	Size (kDa)	Amino acids	Homolog in ***Yersinia***	Features	Localization	Role
PcrG	PA1705	11	98	LcrG (56%)	Binds to PcrV	Bacterial cytosol	Negative regulator
PcrV	PA1706	32.3	294	LcrV (57%)	Cap, Pore	Bacterial Surface	Translocational pore
PcrH	PA1707	18.4	167	LcrH/SycD (76%)	Binds to PopB and PopD	Bacterial cytosol	Chaperone for PopB and PopD
PopB	PA1708	40.1	390	YopB (60%–61%)	Pore	On eukaryotic cell membrane	Translocational pore
PopD	PA1699	31.3	295	YopD (59%–60%)	Pore	On eukaryotic cell membrane	Translocational pore

### Four type III secretory toxins of *P. aeruginosa*

**Figure 4:**
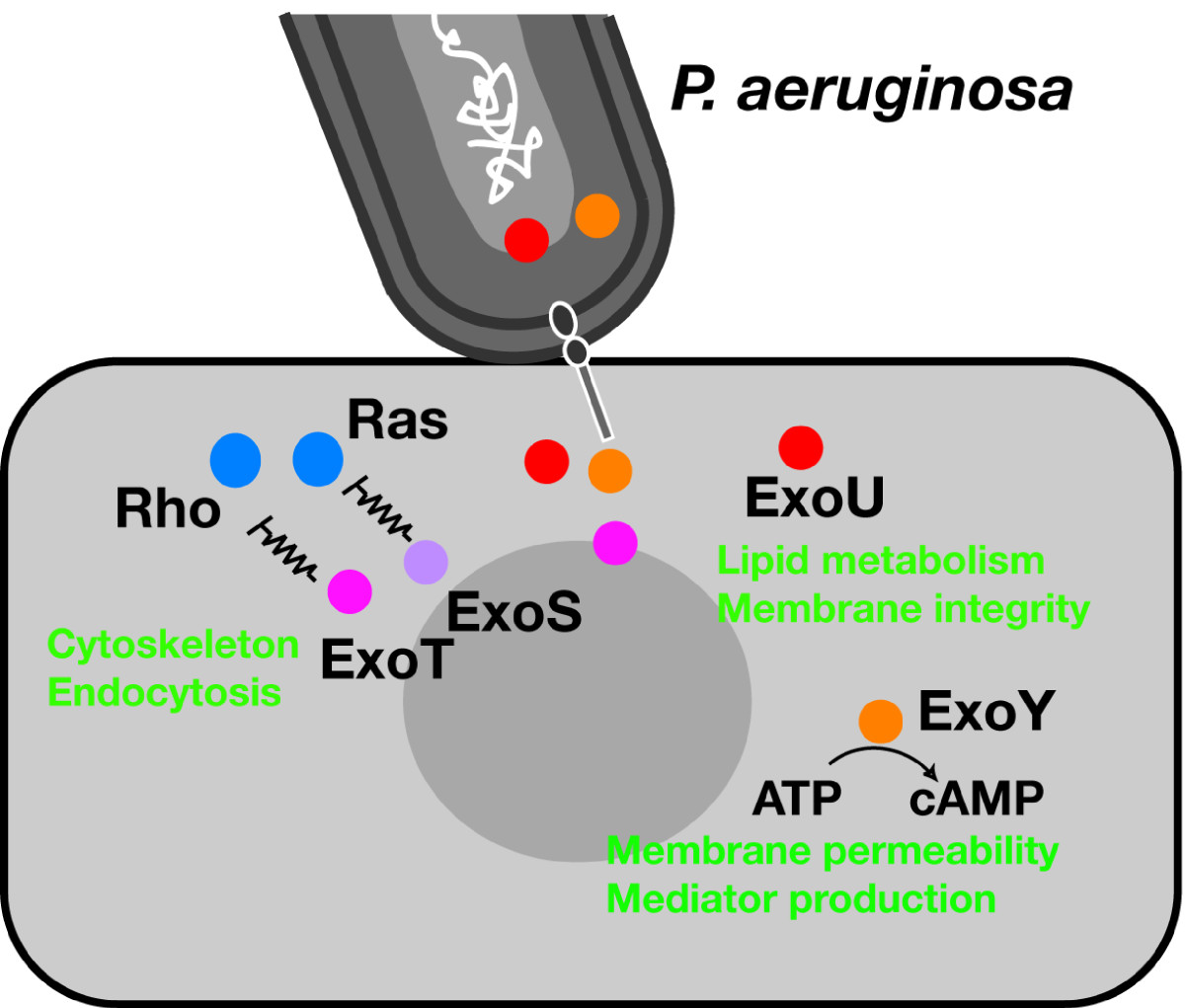
**Contact-dependent toxin translocation during type III secretion in** ***Pseudomonas aeruginosa*** **.** *P. aeruginosa* translocates toxins after direct contact with the surface of the target eukaryotic cell. ExoS and ExoT modulate the cytoskeleton and endocytosis through interaction with Ras and/or Rho GTPases; ExoU disrupts the integrity of the lipid membrane by targeting phospholipids; and ExoY causes edema formation by increasing cyclic adenosine monophosphate.

Till date, *P. aeruginosa* is known to secrete at least four effector molecules (type III secretory toxins) via TTSS: ExoS, ExoT, ExoU, and ExoY (Table 4, Figure 4). The virulence of each strain differs depending on the genotypes and phenotypes of the type III secretory toxins [32, 33, 52].

**Table 4:** ***Pseudomonas aeruginosa*** **type III effector molecules**

Effectors	Other names	Genes	PA number	Protein size	Amino acids	Homologous proteins	Activity	Effect on host
ExoS	49-kDa exoenzyme S	*exoS*	PA3841	49 kDa	453	*Yersinia* YopE	ADP-ribosyltransferase	Antiphagocytosis
						*Salmonella* SptP	GAP activity	
ExoT	53-kDa exoenzyme S	*exoT*	PA0044	53 kDa	457	*Yersinia* YopE	GAP activity	Inhibition of wound healing
	Exoenzyme T					*Salmonella* SptP		
ExoU	PepA	*exoU*	-	72 kDa	687	Mammalian cPLA2	Patatin-like phospholipase	Cell death
						Plant patatins		Acute lung injury
								Bacteremia, sepsis
ExoY	-	*exoY*	PA2191	42 kDa	378	*B. anthoracis* EF	Adenylate cyclase	Edema, inhibition of inflammatory cytokine secretion
						*Bordetella* CyaA

#### ExoS

*P. aeruginosa* exoenzyme S was originally characterized as a toxin distinct from exotoxin A exhibiting ADP-ribosyltransferase activity [17]. Exoenzyme S ADP-ribosylates vimentins and several Ras-related GTP-binding proteins, including Rab3, Rab4, Ral, Rap1A, and Rap2 [67, 68]. The enzymatic reaction requires a soluble eukaryotic protein, termed factor-activating exoenzyme S (FAS), to ADP-ribosylate all substrates [69, 70]. Analysis of several deletion peptides showed that 222 amino acids at the carboxyl terminal of exoenzyme S possessed FAS-dependent ADP-ribosyltransferase activity [69, 70]. Expression of the ADP-ribosyltransferase domain of exoenzyme S is cytotoxic to eukaryotic cells [71].

The amino-terminal domain of exoenzyme S has been characterized as a GTPase-activating protein (GAP) for Rho GTPases [72], suggesting that exoenzyme S is a bifunctional type III secreted cytotoxin [71]. I*n vivo* data indicate that the Rho GAP activity of ExoS stimulates the reorganization of the actin cytoskeleton by inhibiting Rac and Cdc42 and stimulates actin stress fiber formation by inhibiting Rho [73].

#### ExoT

Two immunologically undistinguishable proteins, with apparent molecular sizes of 53- and 49-kDa, co-fractionated with exoenzyme S activity [18]. Later, these two exoenzymes were found to be the products of two different genes [31]. ExoT was found to encode a protein of 457 amino acids, with 75% amino acid homology to ExoS. However, ExoT possessed approximately 0.2% of its ADP-ribosyltransferase activity [74]. ExoT diminishes macrophage motility and phagocytosis, at least in part through disruption of the actin cytoskeleton of eukaryotic cells, and blocks wound healing [75, 76]. Biochemical studies have shown that ExoT is a GAP for RhoA, Rac1, and Cdc42 [77, 78]. These data show that ExoT interferes with the Rho signal transduction pathways, which regulate actin organization, exocytosis, cell cycle progression, and phagocytosis [77, 79].

#### ExoU

In 1997, a novel cytotoxin, ExoU (termed PepA by Hauser et al. [34]), was found to be a major contributory factor to lung injury, and the gene *exoU* was cloned from the cytotoxic PA103 strain. A region downstream of *exoU* was found to encode a specific *Pseudomonas* chaperone for ExoU (SpcU) [80]. In *P. aeruginosa*, ExoU and SpcU are coordinately expressed as an operon controlled at the transcriptional level by ExsA [80]. Acquisition of the expression of *P. aeruginosa* ExoU caused increased bacterial virulence and systemic spread in a mouse model of acute pneumonia [33]. Hauser et al. determined the type III secretion genotypes and phenotypes of isolates cultured from patients with VAP: *in vitro* assays indicated that ExoU most closely linked to mortality in animal models was secreted in detectable amounts *in vitro* by 10 (29%) of the 35 isolates examined [34].

**Figure 5:**
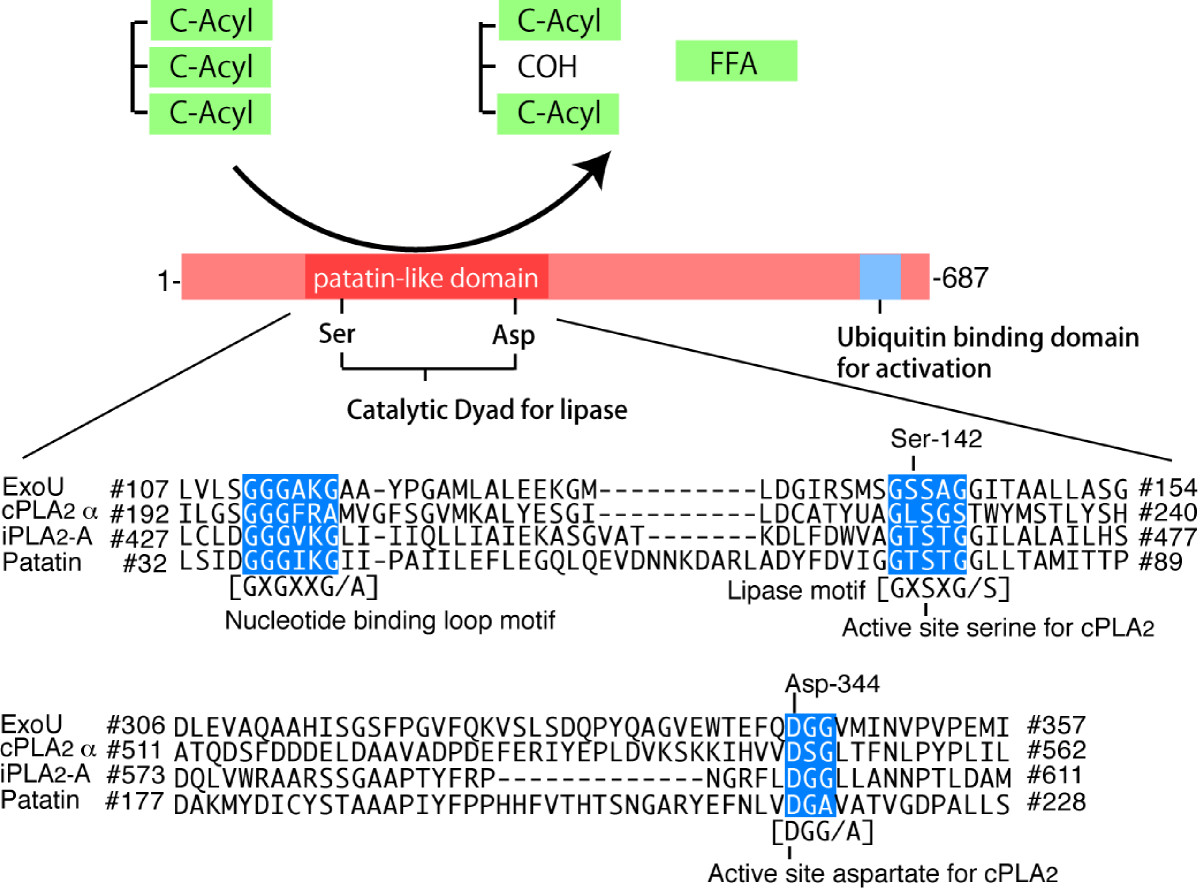
**The molecular structure and functional targets of ExoU.** *P. aeruginosa* ExoU, a major factor causing cytotoxicity and epithelial injury in the lung, contains a patatin domain that catalyzes membrane phospholipids through phospholipase A2 activity. Homology in the amino acid sequence, with a catalytic dyad in the primary structure, was found between patatin, mammalian phospholipase A2 (cPLA2-α and iPLA2), and ExoU. *FFA* free fatty acid.

ExoU has a potato patatin-like phospholipase (PLA) domain (pfam01734 in the Conserved Domain Database of BLAST (National Center for Biotechnology Information, National Library of Medicine, National Institutes of Health, Bethesda, MA, USA); Figure 5). Patatin is a member of a multigene family of vacuolar storage glycoproteins with lipid acyl hydrolase and acyltransferase activities; it represents 40% of the total soluble protein in potato tubers [81]. Sequence alignment of ExoU, potato patatin, and human PLA2 revealed three highly conserved regions in the amino acid sequence of ExoU [82]. In the alignment, Ser-142 and Asp-344 of ExoU corresponded to the catalytic serine and aspartate of PLA2, respectively [82]. Subsequently, using *in vitro* models, it was shown that ExoU exhibits Ser-142- and Asp-344-dependent catalytic PLA2 activity, which requires eukaryotic cell factors for its activation [82, 83]. Then, it was finally concluded that virulent *P. aeruginosa* causes acute lung injury, thereby causing sepsis and mortality, through cytotoxic activity derived from the patatin-like phospholipase domain of ExoU [84]. The cells targeted by ExoU injection through TTSS comprise not only epithelial cells but also macrophages [85]. Through TTSS, ExoU is activated after its translocation into the eukaryotic cell cytosol. It has been recently reported that ubiquitin and ubiquitin-modified proteins are associated with ExoU activation [86, 87].

#### ExoY

ExoY is the fourth type III secretion effector protein controlled by ExoS regulon. ExoY is homologous to the extracellular adenylate cyclases of *Bortedella pertussis* (CyaA), *Bacillus anthracis* (EF), and *Yersinia pestis* (insecticidal toxin) [49]. In assays for adenylate cyclase activity, recombinant ExoY (rExoY) catalyzed the formation of cyclic adenosine monophosphate (cAMP). In contrast to CyaA and EF, rExoY activity was not stimulated or activated by calmodulin. Infection of eukaryotic cells with *P. aeruginosa* producing catalytically active ExoY resulted in the elevation of intracellular cAMP levels and changes in cell morphology [88, 89]. It is more recently reported that ExoY is likely to be a promiscuous nucleotidal cyclase that increases the intracellular levels of cyclic adenosine and guanosine monophosphates, resulting in edema formation [90].

### Epidemiology of the *P. aeruginosa* TTSS

Analysis of type III secretory protein phenotypes was performed in 108 isolates derived from patients with *P. aeruginosa* infections [52]. The mortality rate in patients with *P. aeruginosa* isolates expressing at least one of the type III secretory proteins was 21% compared with the rate of 3% in patients with isolates expressing no type III secretory protein. In another study, infection with isolates secreting TTSS proteins, particularly isolates with an ExoU-positive phenotype, correlated with severe disease [91]. Recently, additional reports have demonstrated an association between the ExoU genotype or phenotype and a poor clinical outcome of *P. aeruginosa* pneumonia. *exoU*-positive isolates were more likely to be fluoroquinolone resistant and exhibit both a *gyrA* mutation and efflux pump overexpression [92]. Clinical isolates containing the *exoU* gene were more likely to be resistant to cefepime, ceftazidime, piperacillin tazobactam, carbapenems, and gentamicin [93]. A fluoroquinolone-resistant phenotype in an ExoU-positive strain contributes to the pathogenesis of *P. aeruginosa* in pneumonia [94]. However, the expression of TTSS exoenzymes in *P. aeruginosa* isolates from bacteremic patients confers a poor clinical outcome, independent of antibiotic susceptibility [95]. Severity of the illness and expression of type III secretory proteins were the strongest predictors of 30-day mortality from *P. aeruginosa* bacteremia [96].

### Update the clinical approach against *P. aeruginosa* pneumonia

*P. aeruginosa* expresses a variety of factors that confer resistance to a broad array of antibacterial agents. Multidrug-resistant *P. aeruginosa* (MDRP) is defined as the resistance to carbapenems, aminoglycosides, and fluoroquinolones. The current increase in the incidence of lethal outbreaks of MDRP is especially a serious concern. Multiple genetic rearrangements, such as chromosomal mutations or horizontal gene transfers (plasmids, integrons, phages), are associated with the acquisition of multidrug resistance in these bacteria. The various mechanisms, such as β-lactamases, carbapenemases or aminoglycoside-modifying enzymes, and mutations in antibiotic targets, efflux pumps, impermeability, are associated in these multidrug resistances. In the management of *P. aeruginosa* pneumonia, the increasing resistance level of these bacteria to most classes of antibacterial agents frequently leads to failure of effective treatment, which is associated with high mortality of the infected patients. Therefore, choosing adequate antibiotics is crucial to increase the survival rate, especially in patients infected with MDRP. Therefore, surveillance in antibiotic resistance must be important to reduce the risk of inadequate antibacterial therapy. In addition, surveillance in TTSSgenotype- and phenotype-associated acute lung injury and sepsis may help to predict the higher risk of lethal outbreaks.

Polymyxin E (colistin) remains the most consistently effective agent against MDPR, while colistin-resistant *P. aeruginosa* has been already reported as a caution of the emergence of pan-resistant strains in the near future [97]. Different strategies against the different targets must be required before the spread of super-resistant strains. Among various experimental therapeutic approaches, the anti-TTSS therapy is reasonable because acute lung injury due to *P. aeruginosa* is highly depending on its TTSS-associated virulence as described above. PcrV has a critical role in the TTS-associated virulence of *P. aeruginosa* as follows [63]. In a series of these studies, active and passive immunization against PcrV in animal models of *P. aeruginosa*-induced lung injury greatly increased survival [63]. Virulent *P. aeruginosa* strains expressing PcrV disabled macrophage phagocytosis. However, antibodies against PcrV blocked this critical antiphagocytic effect [63]. Passive protection with anti-PcrV reduced the inflammatory response, minimized bacteremia, and prevented septic shock in mice and rabbits [98]. The protective capacity of the antibody was Fc-independent as F(ab′)_2_ fragments of polyclonal anti-PcrV were also effective [98]. A murine monoclonal anti-PcrV antibody mAb166 was developed, and its protective effects on acute lung injury were demonstrated when co-instilled with the bacterial challenge or passively transferred to infected animals [99]. The administration of either mAb166 or Fab of mAb166 showed comparable therapeutic effects to rabbit polyclonal anti-PcrV IgG [100]. Based on mAb166, humanized anti-PcrV antibody that was developed by molecular engineering has recently entered phase I/II clinical trials in the USA and Europe for prophylactic and therapeutic uses against *P. aeruginosa* pneumonia in artificially ventilated patients and cystic fibrosis patients [101–103].

## Conclusions

### Summary and future implications

*P. aeruginosa* possesses a sophisticated toxin secretion system to directly inject toxins into the cytosol of target eukaryotic cells. This system, called TTSS, is regulated by the exoenzyme S regulon of *P. aeruginosa*. Through TTSS, *P. aeruginosa* translocates the type III secretory toxins ExoS, ExoT, ExoU, and ExoY. By injecting these toxins into the cytosol of eukaryotic cells, *P. aeruginosa* exploits mammalian enzyme functions to modulate eukaryotic cell signaling.

**Figure 6:**
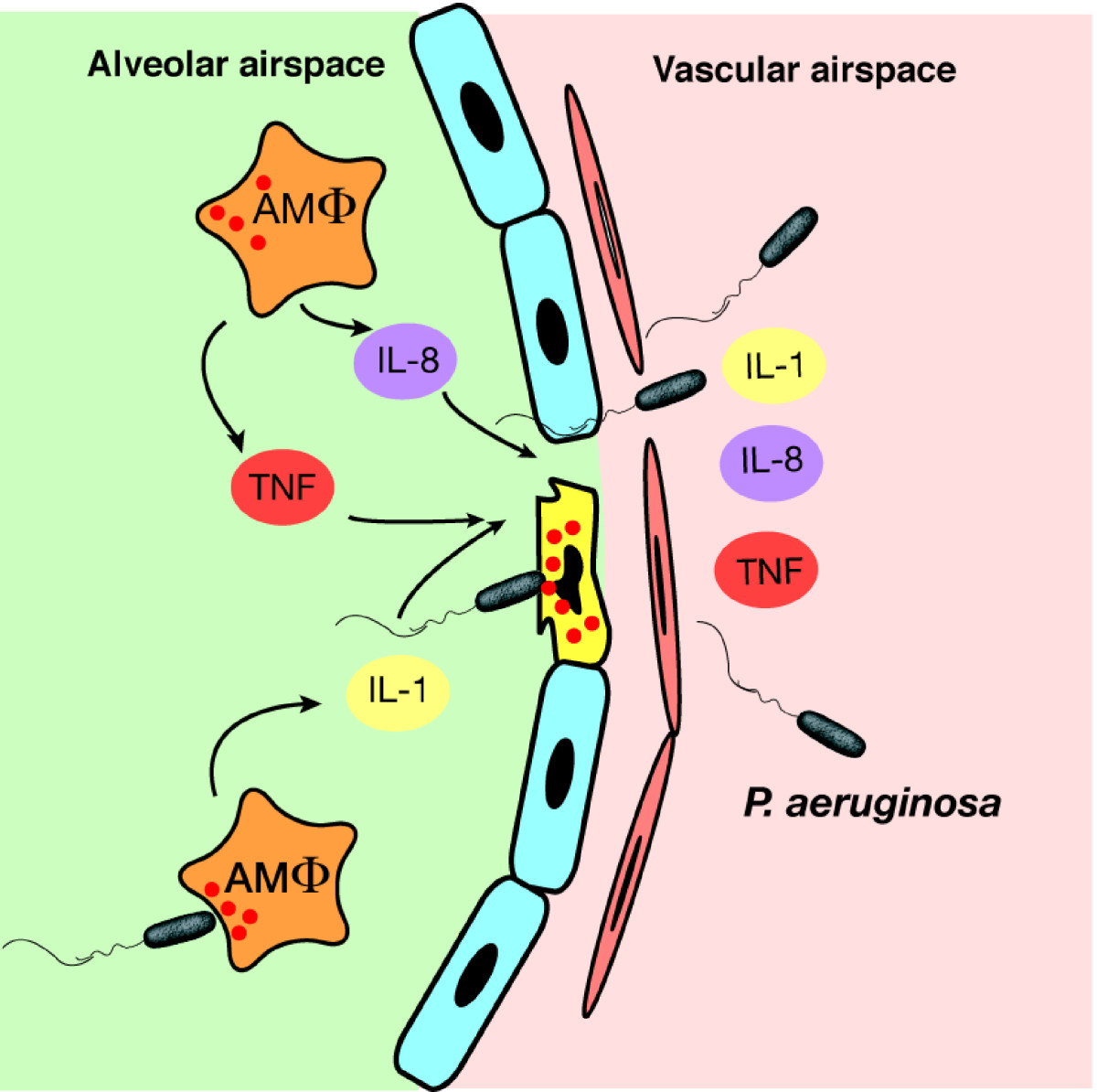
**Acute lung injury caused by** ***Pseudomonas aeruginosa*** **.** *P. aeruginosa* secretes and injects type III secretory toxins (ExoS, ExoT, ExoU, and ExoY) into alveolar macrophages and epithelial cells, blocking macrophage phagocytosis, inducing epithelial disruption, and causing the dissemination of bacterial and inflammatory mediators from the airspace into the systemic circulation, which eventually results in bacteremia and sepsis. *AMΦ* alveolar macrophages, *IL*-*1* interleukin-1, *IL*-*8* interleukin-8, *TNF* tumor necrosis factor.

Of these four toxins, ExoU is the major virulence factor responsible for alveolar epithelial injury in *P. aeruginosa* pneumonia. Virulent strains of *P. aeruginosa* possess the *exoU* gene, whereas nonvirulent strains lack the same. The major pathogenesis of *P. aeruginosa*-induced acute epithelial lung injury and subsequent bacteremia and sepsis is highly dependent on the ExoU phenotype of the strain, while the type III secretory toxins ExoS, ExoT, and ExoY modulate host immunity and cause lung edema (Figure 6). Progress in the field of translational research is now anticipated to prevent the acute lung injury and improve the poor clinical outcome of *P. aeruginosa* pneumonia. What we have learned from our attempts to elucidate the molecular mechanisms underlying acute lung injury over the last 30 years is how well pathogenic bacteria utilize our cell signaling to cause diseases: bacteria know our cell signaling better than we do.

## Authors’ information

TS is a professor in Anesthesiology at Kyoto Prefectural University of Medicine, Japan.

## Authors’ original submitted files for images

Below are the links to the authors’ original submitted files for images. Authors’ original file for figure 1 Authors’ original file for figure 2 Authors’ original file for figure 3 Authors’ original file for figure 4 Authors’ original file for figure 5 Authors’ original file for figure 6

## Abbreviations

ATP: Adenosine triphosphate
cAMP: Cyclic adenosine monophosphate
CF: Cystic fibrosis
cGMP: Cyclic guanosine monophosphate
ExsA: Transcriptional regulator ExsA
FAS: Factor-activating exoenzyme S
GAP: GTPase-activating protein
PAPI: *Pseudomonas aeruginosa* pathogenicity island
TTSS: Type III secretion system
PLA: Patatin-like phospholipase
PLA2: Phospholipase A2
VAP: Ventilator-associated pneumonia.
