# Supplementary figures and images for: The molecular mechanism of acute lung injury caused by Pseudomonas aeruginosa: from bacterial pathogenesis to host response
Source: J Intensive Care. 2014 Feb 18;2:10. doi: 10.1186/2052-0492-2-10 (PMC4267601; doi:10.1186/2052-0492-2-10)

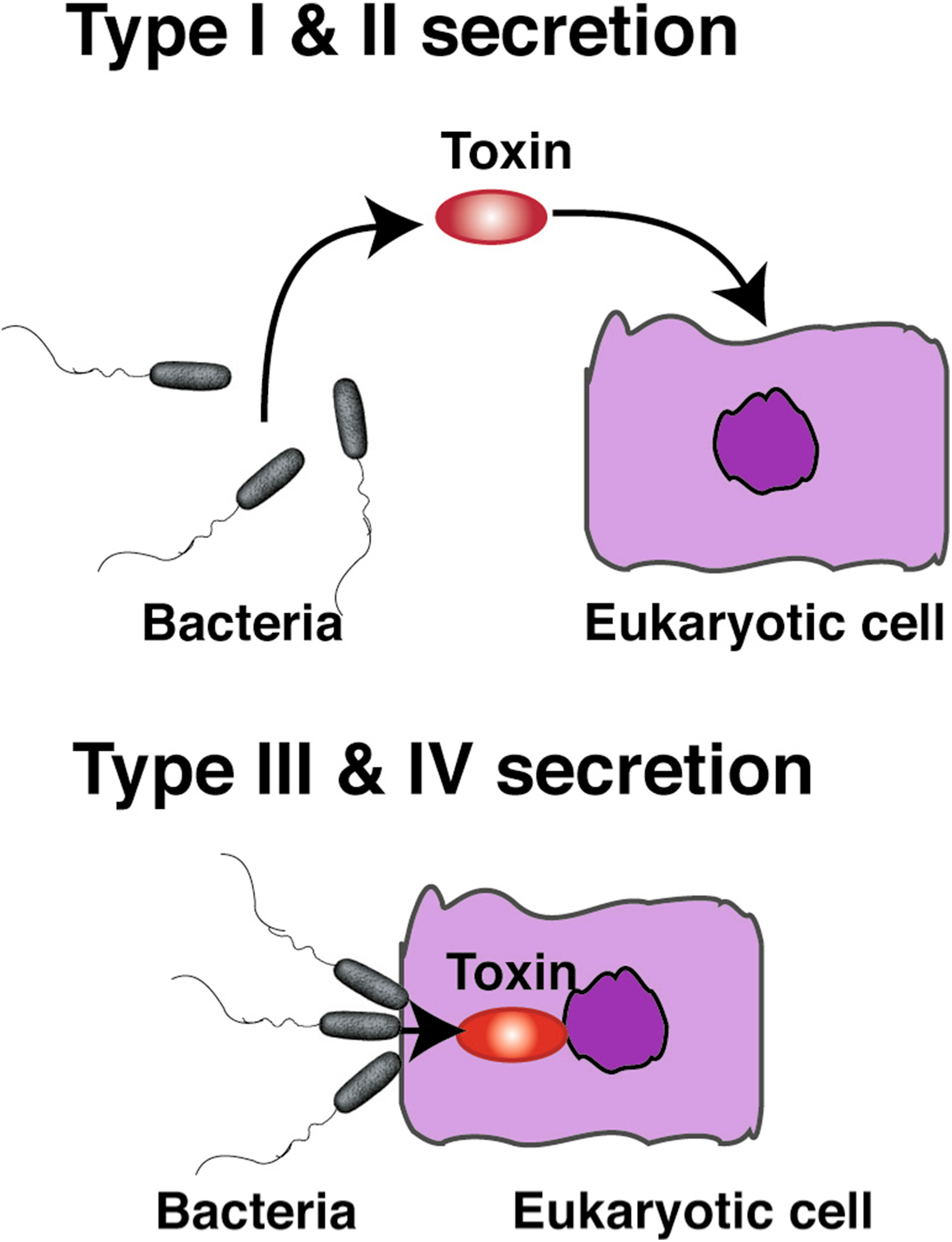

Supplement: Supplementary file 1 — Authors’ original file for figure 1 [file 40560_2013_19_MOESM1_ESM.tif]

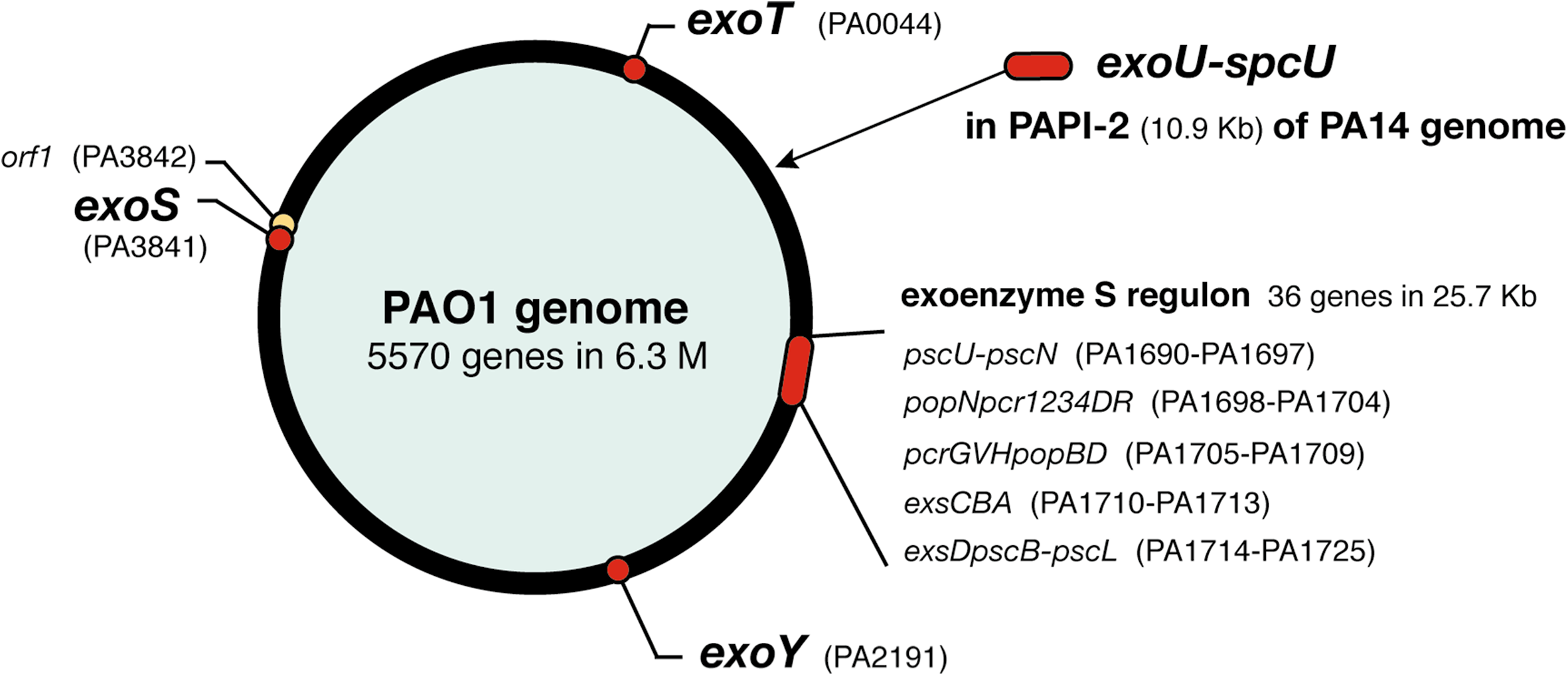

Supplement: Supplementary file 2 — Authors’ original file for figure 2 [file 40560_2013_19_MOESM2_ESM.tif]

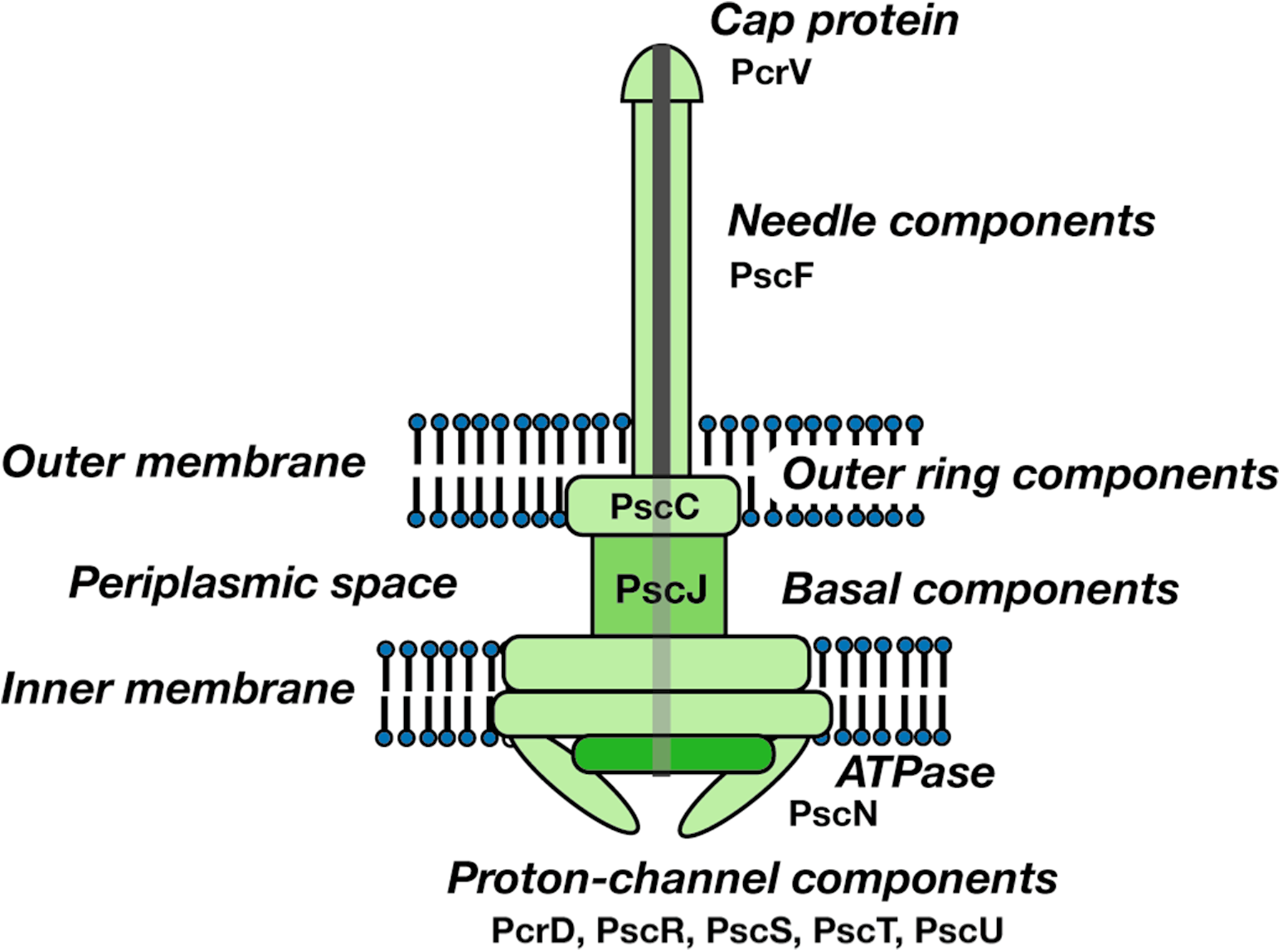

Supplement: Supplementary file 3 — Authors’ original file for figure 3 [file 40560_2013_19_MOESM3_ESM.tif]

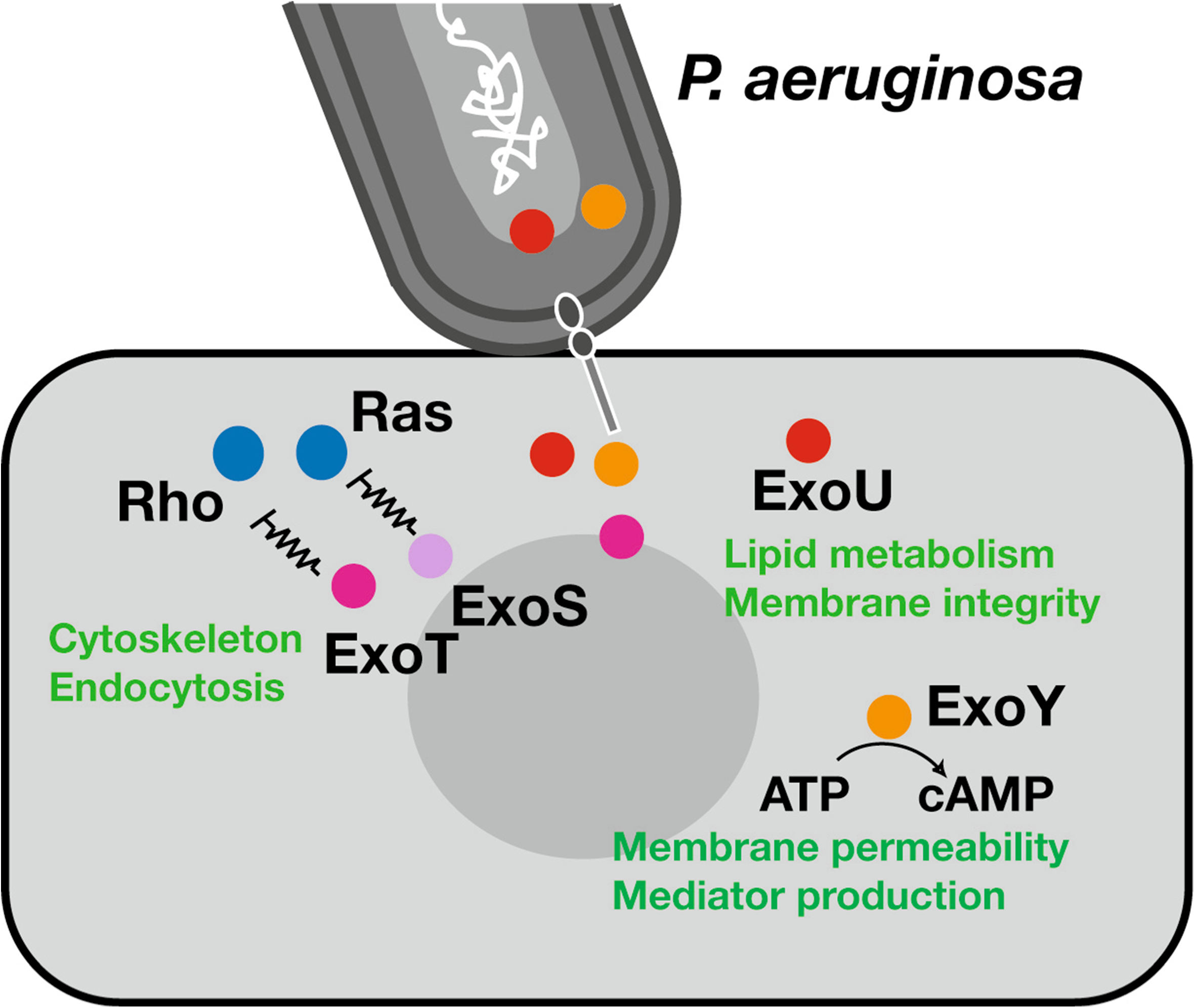

Supplement: Supplementary file 4 — Authors’ original file for figure 4 [file 40560_2013_19_MOESM4_ESM.tif]

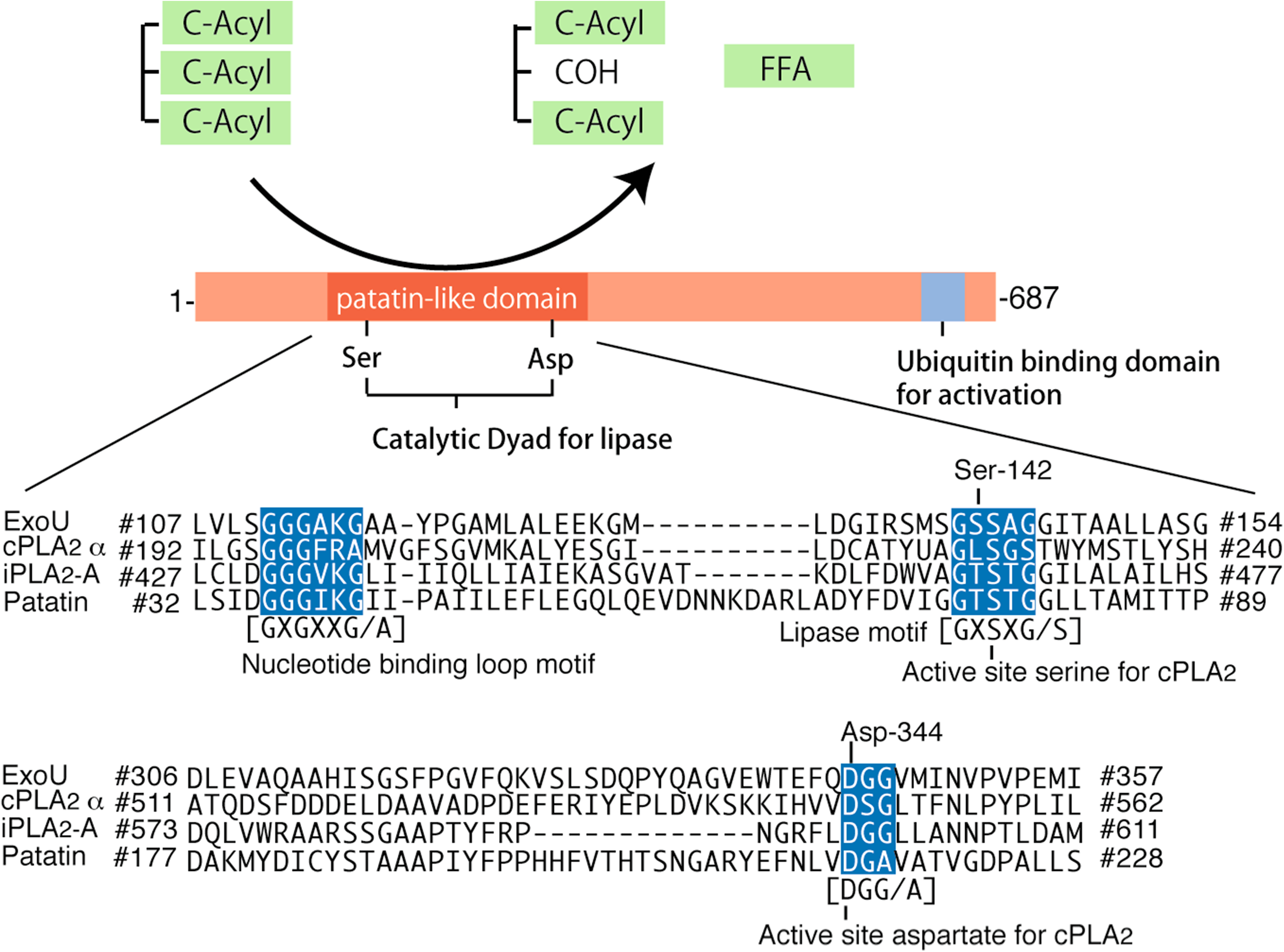

Supplement: Supplementary file 5 — Authors’ original file for figure 5 [file 40560_2013_19_MOESM5_ESM.tif]

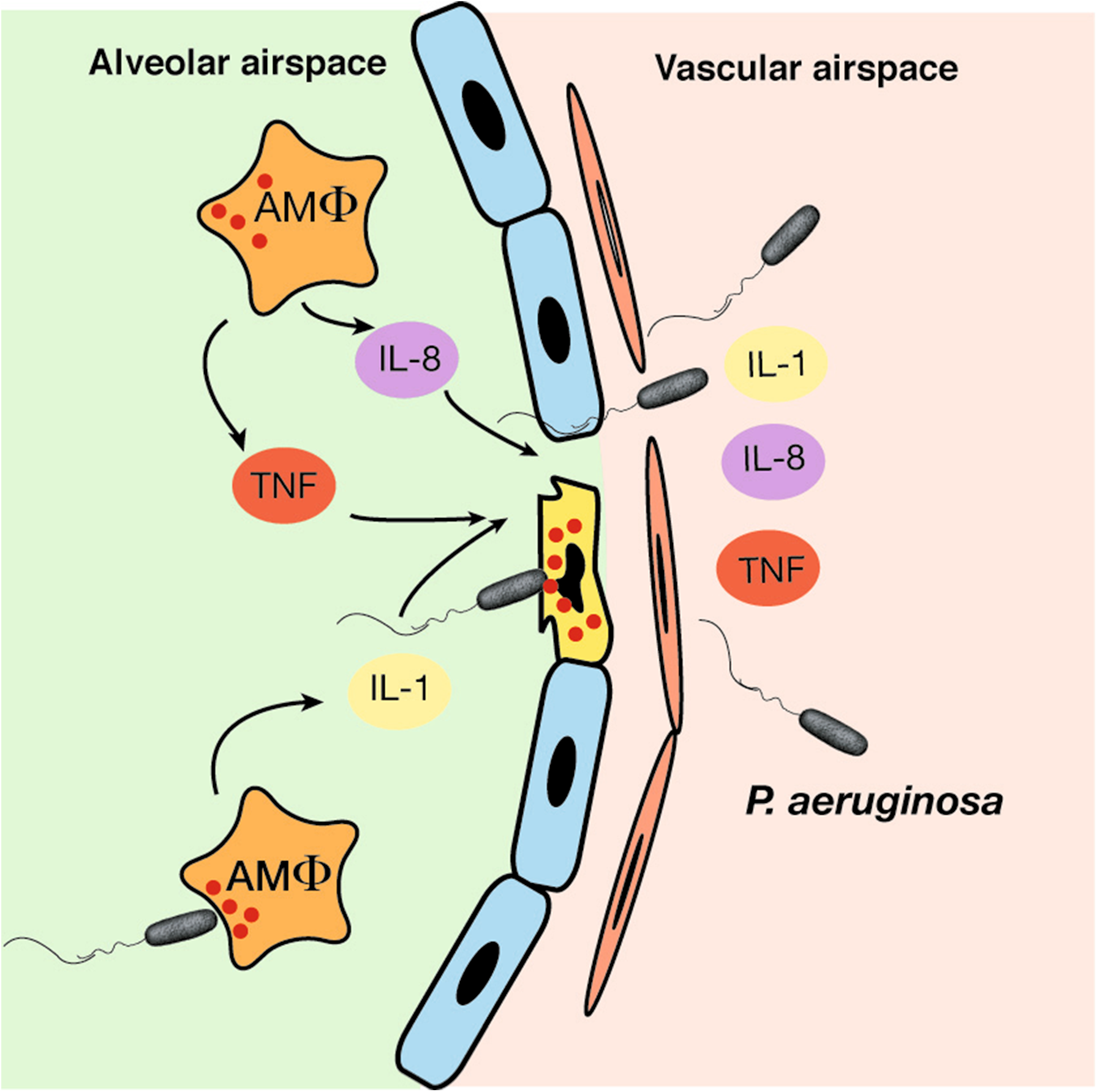

Supplement: Supplementary file 6 — Authors’ original file for figure 6 [file 40560_2013_19_MOESM6_ESM.tif]
